# Artificial Intelligence-assisted Video Colonoscopy for Disease Monitoring of Ulcerative Colitis: A Prospective Study

**DOI:** 10.1093/ecco-jcc/jjae080

**Published:** 2024-06-03

**Authors:** Noriyuki Ogata, Yasuharu Maeda, Masashi Misawa, Kento Takenaka, Kaoru Takabayashi, Marietta Iacucci, Takanori Kuroki, Kazumi Takishima, Keisuke Sasabe, Yu Niimura, Jiro Kawashima, Yushi Ogawa, Katsuro Ichimasa, Hiroki Nakamura, Shingo Matsudaira, Seiko Sasanuma, Takemasa Hayashi, Kunihiko Wakamura, Hideyuki Miyachi, Toshiyuki Baba, Yuichi Mori, Kazuo Ohtsuka, Haruhiko Ogata, Shin-ei Kudo

**Affiliations:** Digestive Disease Center, Showa University Northern Yokohama Hospital, Yokohama, Kanagawa, Japan; Digestive Disease Center, Showa University Northern Yokohama Hospital, Yokohama, Kanagawa, Japan; APC Microbiome Ireland, College of Medicine and Health, University College Cork, Cork, Ireland; Digestive Disease Center, Showa University Northern Yokohama Hospital, Yokohama, Kanagawa, Japan; Department of Gastroenterology and Hepatology, Tokyo Medical and Dental University, Tokyo, Japan; Center for Diagnostic and Therapeutic Endoscopy, Keio University School of Medicine, Tokyo, Japan; APC Microbiome Ireland, College of Medicine and Health, University College Cork, Cork, Ireland; Digestive Disease Center, Showa University Northern Yokohama Hospital, Yokohama, Kanagawa, Japan; Digestive Disease Center, Showa University Northern Yokohama Hospital, Yokohama, Kanagawa, Japan; Digestive Disease Center, Showa University Northern Yokohama Hospital, Yokohama, Kanagawa, Japan; Digestive Disease Center, Showa University Northern Yokohama Hospital, Yokohama, Kanagawa, Japan; Digestive Disease Center, Showa University Northern Yokohama Hospital, Yokohama, Kanagawa, Japan; Digestive Disease Center, Showa University Northern Yokohama Hospital, Yokohama, Kanagawa, Japan; Digestive Disease Center, Showa University Northern Yokohama Hospital, Yokohama, Kanagawa, Japan; Digestive Disease Center, Showa University Northern Yokohama Hospital, Yokohama, Kanagawa, Japan; Digestive Disease Center, Showa University Northern Yokohama Hospital, Yokohama, Kanagawa, Japan; Digestive Disease Center, Showa University Northern Yokohama Hospital, Yokohama, Kanagawa, Japan; Digestive Disease Center, Showa University Northern Yokohama Hospital, Yokohama, Kanagawa, Japan; Digestive Disease Center, Showa University Northern Yokohama Hospital, Yokohama, Kanagawa, Japan; Digestive Disease Center, Showa University Northern Yokohama Hospital, Yokohama, Kanagawa, Japan; Digestive Disease Center, Showa University Northern Yokohama Hospital, Yokohama, Kanagawa, Japan; Digestive Disease Center, Showa University Northern Yokohama Hospital, Yokohama, Kanagawa, Japan; Clinical Effectiveness Research Group, Institute of Health and Society, University of Oslo, OsloNorway; Department of Gastroenterology and Hepatology, Tokyo Medical and Dental University, Tokyo, Japan; Endoscopic Unit, Tokyo Medical and Dental University, Tokyo, Japan; Center for Diagnostic and Therapeutic Endoscopy, Keio University School of Medicine, Tokyo, Japan; Clinical Medical Research Center, International University of Health and Welfare, Narita, Japan; Center for Diagnostic and Therapeutic Endoscopy, San-no Medical Center, Tokyo, Japan; Digestive Disease Center, Showa University Northern Yokohama Hospital, Yokohama, Kanagawa, Japan

**Keywords:** Endoscopic remission, Mayo endoscopic subscore, computer-aided diagnosis

## Abstract

**Backgrounds and Aims:**

The Mayo endoscopic subscore [MES] is the most popular endoscopic disease activity measure of ulcerative colitis [UC]. Artificial intelligence [AI]-assisted colonoscopy is expected to reduce diagnostic variability among endoscopists. However, no study has been conducted to ascertain whether AI-based MES assignments can help predict clinical relapse, nor has AI been verified to improve the diagnostic performance of non-specialists.

**Methods:**

This open-label, prospective cohort study enrolled 110 patients with UC in clinical remission. The AI algorithm was developed using 74 713 images from 898 patients who underwent colonoscopy at three centres. Patients were followed up after colonoscopy for 12 months, and clinical relapse was defined as a partial Mayo score > 2. A multi-video, multi-reader analysis involving 124 videos was conducted to determine whether the AI system reduced the diagnostic variability among six non-specialists.

**Results:**

The clinical relapse rate for patients with AI‐based MES = 1 (24.5% [12/49]) was significantly higher [log-rank test, *p *= 0.01] than that for patients with AI‐based MES = 0 (3.2% [1/31]). Relapse occurred during the 12-month follow-up period in 16.2% [13/80] of patients with AI‐based MES = 0 or 1 and 50.0% [10/20] of those with AI‐based MES = 2 or 3 [log-rank test, *p *= 0.03]. Using AI resulted in better inter- and intra-observer reproducibility than endoscopists alone.

**Conclusions:**

Colonoscopy using the AI-based MES system can stratify the risk of clinical relapse in patients with UC and improve the diagnostic performance of non-specialists.

## Graphical Abstract

**Graphical Abstract:**
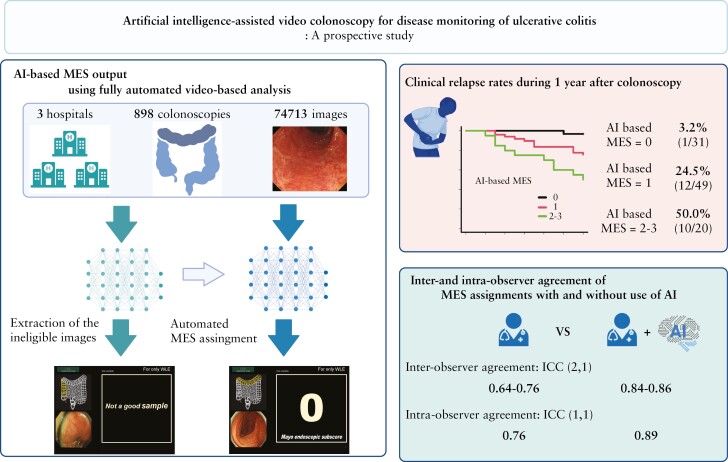

## 1. Intrduction

Ulcerative colitis [UC] is a chronic inflammatory bowel disease that alternates between remission and relapse states.^1^ Maintaining endoscopic remission [ER] for as long as possible using appropriate drug interventions is important because it contributes to long-term clinical remission, lessens the risk of undergoing surgery because of inflammation relapse, and reduces the incidence of colitis-related cancer.^2,3^

With recent implementation of the treat-to-target strategy in clinical practice, there is consensus regarding the significance of achieving ER as a pivotal, long-term therapeutic goal.^4,5^ The Mayo endoscopic subscore [MES]^6^ is the most popular measure of ER [often defined as MES = 0 or 1].^2^ Several recent reports have suggested that patients with MES = 1 have higher relapse and colectomy rates than those with MES = 0.^7,8^ Hence, MES = 0 is currently defined as complete ER to distinguish it from MES = 1. However, a significant challenge arises from the limited agreement among examiners, particularly non-specialists.^9–11^

Artificial intelligence [AI] has been reported to provide precise outputs and has gained considerable attention as a potential solution to variability among endoscopists.^10,12–17^ Nonetheless, no study has been conducted to ascertain whether AI-based MES assignments can help predict clinical relapse, nor has computer-aided diagnosis [CAD] been verified to change the diagnostic performance of non-specialists.

This prospective study assessed the prognostic efficacy of AI-based MES assignments for patients in clinical remission from UC. It also assessed whether use of AI-driven CAD improves the diagnostic performance of non-specialists.

## 2. Methods

### 2.1. Study design and sites

We conducted a single-group, open-label, prospective study. Patient enrolment and colonoscopy procedures were conducted at a single centre [Showa University Northern Yokohama Hospital]. The CAD system was trained using endoscopic data from three centres [Keio University, Tokyo Medical and Dental University, and Showa University Northern Yokohama Hospital].

### 2.2. AI-based MES output using fully automated video-based analysis

**Figure 1:**
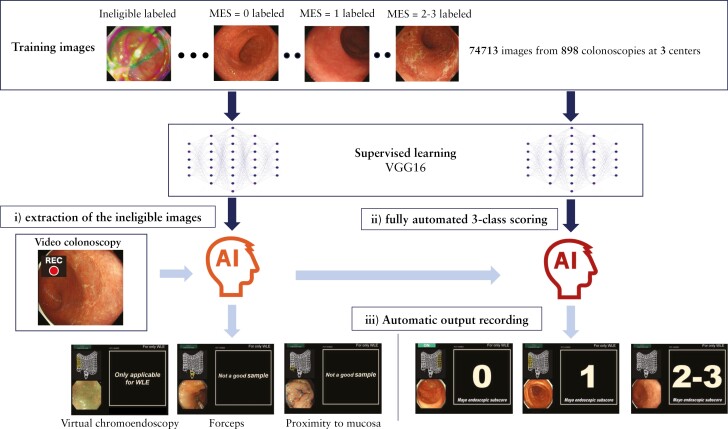
Artificial intelligence [AI]-based Mayo endoscopic subscore [MES] diagnostic system. The system has three functions: i] real-time output of ‘not a good sample’ to inform endoscopists that the image is ineligible for analysis in cases of halation, inadequate air delivery, proximity to mucosa, chromoendoscopy, and virtual chromoendoscopy; ii] fully automated three-class MES output; and iii] recording of an endoscopic image and AI output.

The AI system [EB-UC2 prototype; Cybernet Systems, Tokyo, Japan] used in this study is a fully automated, video-based system that provides a three-class MES output [0, 1, and 2 or 3]. This system has three functions: i] real-time output of ‘not a good sample’ to inform endoscopists that the image is ineligible for analysis in cases of halation, inadequate air delivery, proximity to mucosa, chromoendoscopy, and virtual chromoendoscopy [ie, narrow-band imaging]; ii] fully automated three-class MES output based on video analysis; and iii] recording of endoscopic images and AI output when the endoscopist pushes a button on the left-side handle of the colonoscope [Figure 1; see Supplementary Data 1 and 2].

The development of our AI system leveraged the power of a convolutional neural network. The chosen architectural framework was the 16-layer Visual Geometry Group [VGG16] network. To train the AI algorithm, 74 713 frames were collected from 898 patients who underwent colonoscopies at three centres between October 2016 and September 2021. Of these, 49 872 frames were used to develop a fully automated, three-class scoring output algorithm. Each frame was labelled with an MES by two experienced endoscopists [NO and YaM]. In cases of diagnostic discrepancies between two endoscopists, the final diagnosis was determined through discussion with another endoscopist [KO]. The remaining 24 841 frames were used to develop the algorithm for real-time output of ‘not a good sample’.

### 2.3. Participants

Patients were considered eligible for the study, if they had been diagnosed with UC, who had sustained clinical remission [partial Mayo score ≤ 2] for at least 6 months and had undergone regular colonoscopies for monitoring of disease severity and surveillance of dysplasia. Eligible patients were enrolled between December 2021 and July 2022 at Showa University Northern Yokohama Hospital. Exclusion criteria included patients with active UC [partial Mayo score > 2], a history of colon surgery, and those receiving prednisolone in the 6 months before colonoscopy. Patients with UC in clinical remission underwent colonoscopy using the AI system, and were observed for 12 months after colonoscopy.

### 2.4. Colonoscopy procedure

We used commercially available colonoscopes [CF-HQ290ZI, PCF-H290ZI, PCF-PQ260I/L, CF-XZ1200I, and CF-EZ1500DI; Olympus Medical Systems, Tokyo, Japan]. For bowel preparation, patients ingested 2–4 L polyethylene glycol solution on the morning of the examination, and clearance of intestinal fluid was verified before initiating the colonoscopy, which was conducted under conscious sedation with intravenous diazepam [5–10 mg] or midazolam [2–10 mg].

The endoscopist assigned the MES in five colorectal segments [caecum-ascending colon, transverse colon, descending colon, sigmoid colon, and rectum] while withdrawing the colonoscope from the caecum. At least one image of the most severely inflamed region was taken in each segment to obtain the diagnostic AI output.

Patients were classified in one of three groups on the basis of the AI‐based MES for the worst segment. In scoring each segment, because multiple endoscopic images were taken for each segment, the AI predictions were based on the most frequently obtained score [eg, if there were three AI‐based MES = 0 and five AI‐based MES = 1 outputs, the segment was deemed to be AI‐based MES = 1]; if the numbers of each output were the same, the diagnosis with the highest total probability was adopted.

### 2.5. Registration of case report forms

After each colonoscopy, AI predicted the inflammatory response values for each image output and recorded them in a comma-separated file. One endoscopist [MM], who was not informed of the patient’s clinical course or endoscopic information, then aggregated the AI output from the comma-separated file and registered AI-based MES assignments. Both patients and the clinicians were blinded to the AI output. Another expert endoscopist [NO] registered each patient’s characteristics and clinical course data in the database.

### 2.6. Outcome measures

Patients were classified as AI‐based MES = 0, AI‐based MES = 1, or AI‐based MES = 2 or 3. They were seen in the outpatient clinic every 8 or 12 weeks for 12 months or until clinical relapse, defined as a partial Mayo score > 2. After colonoscopy, medical intervention during visits to outpatient clinics followed Japanese clinical practice guidelines.^18^ The primary outcome measure was the rate of clinical relapse of AI-based MES = 0 vs AI-based MES = 1 patient within 12 months after colonoscopy. The secondary outcome measure was the rate of clinical relapse of AI-based MES = 0 or 1 vs AI-based MES = 2 or 3 patients within 12 months after colonoscopy.

To compare AI-based predictions with human-based predictions of future clinical relapse, receiver operating characteristic [ROC] curves were constructed using the corresponding MES assignments, and the corresponding area under the curve [AUC] values were computed to evaluate AI- and human-based MES performance.

The ability of AI to identify images appropriate for scoring, ER [MES = 0 or 1], and complete ER [MES = 0] were assessed. The ground truth labels were applied to each image by two specialist endoscopists.

A multi-video, multi-reader sub-analysis was conducted to determine whether this system enhances diagnostic accuracy and reduces inter- and intra-examiner variabilities among non-specialists [see Supplementary Material for details of selection and creation of video].

Six non-specialists viewed each of the 124 videos, ranging in length from 5 to 10 s, four times and assigned an MES for each one. During the initial two viewings [sessions 1 and 2] of the endoscopic video, they were blinded to the AI outputs. During the latter two viewings [sessions 3 and 4], they monitored both the endoscopic videos and the AI output. A minimum interval of 2 weeks was maintained between each session. Finally, differences in diagnostic accuracy and inter- and intra-examiner agreement were evaluated with and without AI. The results of sessions 1 and 3 were used to assess diagnostic accuracy, and the results of sessions 1–4 were used to evaluate inter- and intra-examiner variability.

### 2.7. Sample size calculation

We estimated the required sample size based on a previous study conducted at the same hospital.^19^ An MES = 0/MES = 1/MES = 2 or 3 enrollment ratio of 2:3:1 was based on experience in our hospital. We assumed a 12-month clinical relapse rate of 5% for patients with MES = 0 and 25% for patients with MES = 1.^18^ With a two-sided significance of 0.05 and a power of 80%, 35 patients with MES = 0 and 52 with MES = 1 are required to measure the primary outcome. Additionally, at least 18 patients with MES = 2 or 3 are required for the secondary outcome. We estimated that 110 participants were needed to allow for patient dropout.

### 2.8. Statistical analysis

A per-protocol analysis that excluded patients with missing data was used for the primary endpoint. All statistical analyses were performed using R [v. 2.13.0; www.R-project.org]. Numerical data and continuous variables were expressed as a median (interquartile range [IQR]] or mean, 95% confidence interval [CI]), and statistical significance was assessed using Fisher’s exact test, McNemar’s test, Student’s t test, or the Mann–Whitney U test, as appropriate. Kaplan–Meier analysis and the log-rank test were used to analyse the cumulative relapse-free rate. Bonferroni correction was used when multiple comparisons were performed. Multivariate analysis was performed using unconditional logistic regression with clinical recurrence as the dependent variable and endoscopic finding as the independent variable. Intra- and inter-examiner agreements were assessed using the intraclass correlation coefficient. A two-sided value of *p *< 0.05 was considered statistically significant.

### 2.9. Research enrolment and ethics

All patients provided informed consent for the procedures and study participation. The Ethics Committee of Showa University Northern Yokohama Hospital approved the study protocol [No. 21H0003]. The study was registered in the clinical trial registry of the University Hospital Medical Information Network [UMIN000048158] and conducted by the guidelines of the Declaration of Helsinki. All authors had access to the study data and reviewed and approved the final manuscript.

## 3. Results

**Figure 2:**
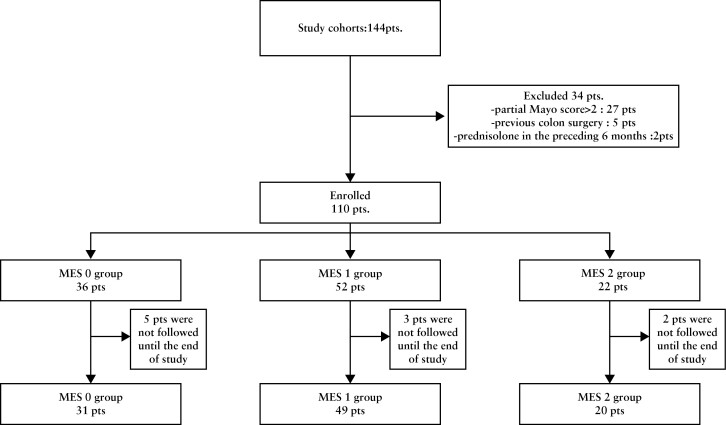
Flowchart of patient enrolment.

A flowchart of the patient inclusion process is shown in Figure 2. Of 144 patients with UC screened for eligibility, 34 were ineligible because they had a partial Mayo score > 2, had used corticosteroids in the previous 6 months, or had previous colon surgery. The remaining 110 patients were included in the study and underwent AI-assisted colonoscopy; 36 patients were classified as AI-based MES = 0, 52 as AI-based MES = 1, and 22 as AI-based MES = 2 or 3. Of these, five patients with AI-based MES = 0, three with AI-based MES = 1, and two with AI-based MES = 2 or 3 dropped out during the 12-month follow-up period. We therefore analysed 31 patients with an AI-based MES = 0, 49 patients with AI‐based MES = 1, and 20 patients with AI‐based MES = 2 or 3 [Figure 2]. At the outpatient visit following colonoscopy, 31 patients with AI-based MES = 0 received no additional treatment; six of 49 patients with AI-based MES = 1 received some additional treatment [three received temporally additional rectal budesonide, two received additional rectal 5-aminosalicylic acid suppository, and one received an increased dose of oral 5-aminosalicylic acid]; and seven of 20 patients with AI-based MES = 2 or 3 also received additional treatment [three received an increased dose of oral 5-aminosalicylic acid, two received temporally additional rectal budesonide, and two received additional rectal 5-aminosalicylic acid suppository] [Table 1].

**Table 1:** Patient characteristics.

	AI-based MES = 0 [*N* = 31]	AI-based MES = 1 [*N* = 49]	AI-based MES = 2 or 3 [*N* = 20]	*p*-value
Male, *n* [%]	15 [48.4]	27 [55.1]	10 [50]	0.99
Age [y], median [IQR]	53.1 [22–79]	54.7 [18–82]	43.6 [16–61]	0.48
Duration of disease [y], median [IQR]	11.5 [1–26]	12.5 [1–47]	12.3 [1–29]	0.30
Extent of disease, *n* [%]				0.08
Extensive colitis	16 [51.6]	38 [77.6]	10 [50]	
Left-sided colitis	7 [22.6]	6 [12.2]	6 [30]	
Proctitis	8 [25.8]	5 [10.2]	4 [20]	
Concomitant therapy, *n* [%]				
Oral 5-aminosalicylic acid	30 [96.8]	42 [85.7]	16 [80]	0.72
Topical 5-aminosalicylic acid	4 [12.9]	9 [18.4]	8 [40]	0.07
Immunomodulator	5 [16.1]	11 [22.4]	3 [15]	0.74
Biologic agent	5 [16.1]	9 [18.4]	6 [30]	0.34
Serum evaluation, median [IQR]				
White blood cell count [g/μL]	5310 [2560–7670]	5450 [2610–9470]	5830 [3230–12 410]	0.94
Haemoglobin [g/dL]	14.1 [8.6–17.7]	14.2 [8.9–17.4]	13.8 [12.2–16]	0.55
Albumin [g/dL]	4.6 [3.9–5.3]	4.5 [3.7–5.2]	4.6 [3.8–5.6]	0.49
C-reactive protein [mg/dL]	0.11 [0.03–0.71]	0.07 [0.04–0.28]	0.07 [0.04–0.22]	0.98
Erythrocyte sedimentation rate [mm/h]	9.9 [2–37]	9.4 [1–37]	8.8 [2–32]	0.85

IQR, interquartile range; AI, artificial intelligence, MES, Mayo Endoscopic Subscore; y, years.

### 3.1. Clinical relapse rates of AI-based MES = 0 and MES = 1 patients [primary outcome]

**Figure 3:**
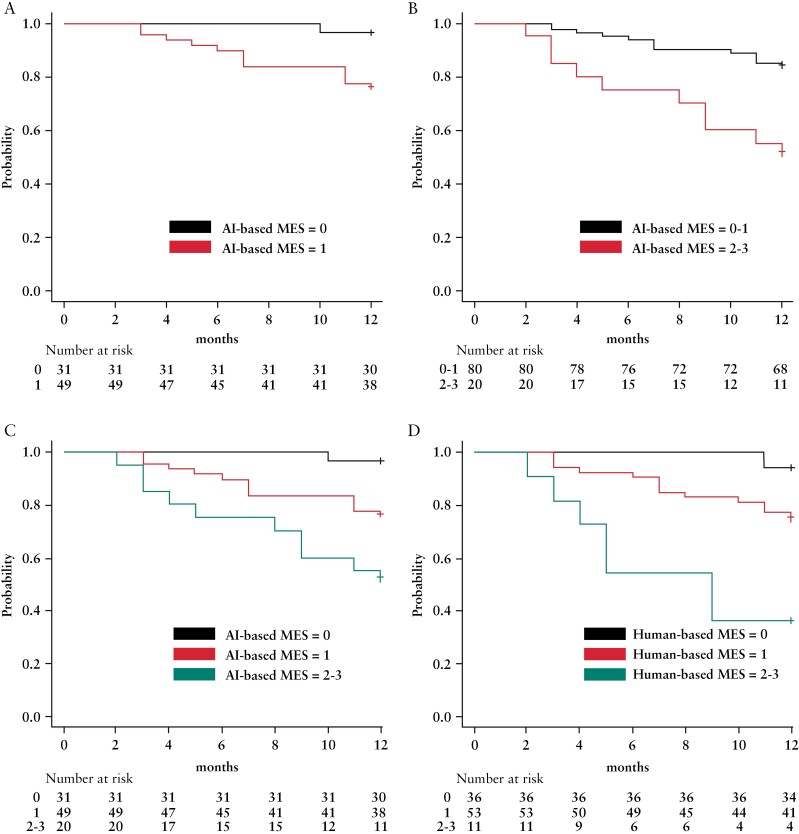
Kaplan–Meier estimates of proportions of all patients free from clinical relapse based on AI outputs. [a] Patients with an AI-based MES = 0 vs those with an AI-based MES = 1 [log-rank test: *p *< 0.01]. [b] Patients with an AI-based MES = 0 or 1 vs those with an AI-based MES = 2 or 3 [log-rank test: *p *= 0.013]. [c] Patients with an AI-based MES = 0 vs those with an AI-based MES = 1 vs those with an AI-based MES = 2 or 3 [log-rank test: *p *< 0.01]. [d] Patients with a human-based MES = 0 vs those with a human-based MES = 1 vs those with a human-based MES = 2 or 3 [log-rank test: *p *< 0.01].

Among patients with AI-based MES = 0 or 1, per-protocol analysis revealed that relapse occurred during the 12-month follow-up period in 3.2% [1/31] [95% CI, 0.1%–16.7%] of patients with AI-based MES = 0, and 24.5% [12/49] [95% CI, 13.3%–38.9%] of those with AI-based MES = 1. The clinical relapse rate for patients with AI-based MES = 1 was significantly higher than that for patients with AI-based MES = 0 [*p* < 0.01; Table 2]. Kaplan–Meier curves showing the cumulative probabilities of being relapse-free in the two groups are shown in Figure 3a [log-rank test: *p *= 0.01].

**Table 2:** Sustained clinical remission rate during 12 months after colonoscopy.

	AI-based MES = 0	AI-based MES = 1	*p-*value
Patients limited with AI-based MES = 0 or 1 [*n* = 80]	3.2 [1/31] [0.1–16.7]	24.5 [12/49] [13.3–38.9]	0.01
Patients with any AI-based MES [*n* = 100]	16.2 [13/80] [8.9–26.2]	50.0 [10/20] [27.2–72.8]	0.03

Values given as % [number of patients with clinical relapse during 12 months after colonoscopy/total number of patients classified with corresponding AI-based MES] [95% confidence interval].

MES, Mayo Endoscopic Subscore.

### 3.2. Clinical relapse rates of AI-based MES = 0 or 1 and MES = 2 or 3 patients [secondary outcome]

Per-protocol analysis revealed that relapse occurred during the 12-month follow-up period in 16.2% [13/80] [95% CI, 8.9%–26.2%] of patients with AI‐based MES = 0 or 1 and 50.0% [10/20] [95% CI, 27.2%–72.8%] of those with AI‐based MES = 2 or 3. The clinical relapse rate for patients with AI‐based MES = 0 or 1 was significantly lower than that for patients with AI‐based MES = 2 or 3 [*p *= 0.03; Table 2]. Kaplan–Meier curves showing the cumulative probabilities of being relapse-free in the two groups are shown in Figure 3b [log-rank test: *p *< 0.01]. Kaplan–Meier curves showing the cumulative probabilities of being relapse-free in the AI‐based MES = 0, AI‐based MES = 1, and AI‐based MES = 2 or 3 groups are also shown in Figure 3c [log-rank test: *p *= 0.01].

### 3.3. Comparison of AI- and human-based MESs in prediction of future clinical relapse

Kaplan–Meier curves showing the cumulative probabilities of being relapse-free in the human‐based MES = 0, human‐based MES = 1, and human‐based MES = 2 or 3 groups are shown in Figure 3d [log-rank test: *p *< 0.01].

**Figure 4:**
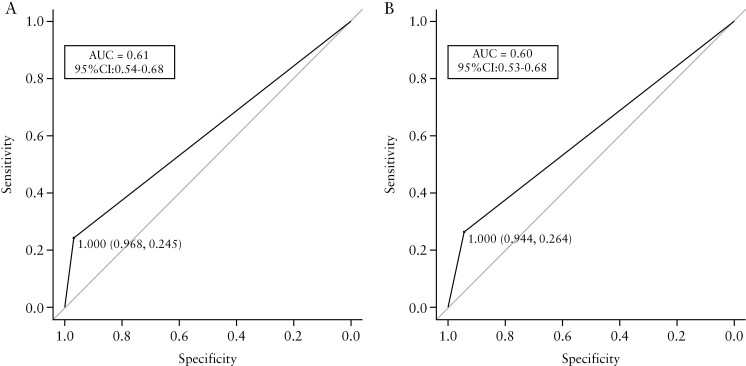
Receiver operating characteristic curves showcasing multiple criteria. The areas under the curve [AUC] for predicting clinical relapse after 12 months were 0.61 and 0.60 for [a] AI-based MES and [b] human-based MES, respectively.

ROCs for AI- and human-based MESs are shown in Figure 4a and b. The individual AUC values for predicting clinical relapse after 12 months were 0.61 and 0.60 for the AI- and human-based MESs, respectively.

### 3.4. Diagnostic performance of the AI-based algorithm

A total of 11 472 images was taken from 110 UC patients who enrolled in this study. AI was able to identify images appropriate for scoring with 83.0% [95% CI, 81.8%–84.2%] sensitivity, 86.3% [95% CI, 85.5%–87.0%] specificity, and 85.1% [95% CI, 84.4%–85.8%] accuracy.

Among 4395 of the above images, AI identified ER with 96.9% [95% CI, 96.2%–97.4%] sensitivity, 78.4% [95% CI, 75.5%–81.2%] specificity, and 93.4% [95% CI, 92.6–94.1%] accuracy. AI identified complete ER with 93.8% [95% CI, 92.8%–94.7%] sensitivity, 77.2% [95% CI, 75.2%–79.2%] specificity, and 87.1% [95% CI, 86.1%–88.1%] accuracy [Table 3].

**Table 3:** Diagnostic ability of the AI-based MES system.

Diagnosis	Sensitivity	Specificity	Accuracy	PPV	NPV
Image appropriate for scoring [*n* = 11,472]	83.0 [81.8–84.2] [3378/4069]	86.3 [85.5–87.0] [6386/7403]	85.1 [84.4–85.8] [9764/11,472]	76.9 [75.6–78.1] [3378/4395]	90.2 [89.5–90.9] [6386/7077]
Endoscopic remission [*n* = 4395]	96.9 [96.2–97.4] [3453/3565]	78.4 [75.5–81.2] [651/830]	93.4 [92.6–94.1] [4104/4395]	95.1 [94.3–95.8] [3453/3565]	85.3 [82.6–87.8] [651/763]
Complete endoscopic remission [*n* = 4395]	93.8 [92.8–94.7] [2462/2626]	77.2 [75.2–79.2] [1366/1769]	87.1 [86.1–88.1] [3828/4395]	85.9 [84.6–87.2] [2462/2865]	89.3 [87.6–90.8] [1366/1530]

Values given as % [95% confidence interval] [*n*/*N*].

MES, Mayo Endoscopic Subscore; PPV, positive predictive value; NPV, negative predictive value.

### 3.5. Clinical effect of AI on diagnostic performance of non-specialist endoscopists

The diagnostic value of AI-based MES was significantly higher than that of non AI-based MES [McNemar test, *p* < 0.01; see Supplementary Tables S1 and S2]. Intraclass correlation coefficients among six non-specialists showed that use of AI improved inter-observer reproducibility: 0.64 [session 1] and 0.76 [session 2] without AI; and 0.86 [session 3] and 0.84 [session 4] with AI. Similarly, intra-observer reproducibility using AI was higher than that without using AI [intraclass correlation coefficients: 0.76 without AI and 0.89 with AI] [Table 4].

**Table 4:** Inter- and intra-observer agreement among six non-expert MES assignments with and without use of AI.

Inter-observer agreement among six trainees				
Viewing session	Use of AI	ICC [2,1]	95% CI	*p-*value
1	Without	0.64	0.57–0.71	<0.01
2	Without	0.76	0.72–0.82	<0.01
3	With	0.86	0.83–0.90	<0.01
4	With	0.84	0.8–0.88	<0.01

Intra-observer agreement between two readings				
Viewing session	Use of AI	ICC [1,1]	95% CI	*p* value
1**–**2	Without	0.76	0.73–0.79	<0.01
3**–**4	With	0.89	0.87–0.90	<0.01

MES, Mayo Endoscopic Subscore; AI, artificial intelligence; ICC, intraclass correlation coefficient; CI, confidence interval.

## 4. Discussion

To the best of our knowledge, this is the first study to assess whether an AI-based MES can stratify the risk of future clinical relapse of UC patients in clinical remission and how CAD influences the diagnostic MES assignments by non-specialists. One strength of our study is that it achieves effective relapse risk stratification based on three classifications used in routine clinical practice.

With the emergence of convolutional neural networks, AI-assisted colonoscopy has garnered significant attention as a novel disease-monitoring tool to assess inflammation severity objectively and accurately. Although such a system is expected to offer a precise and objective diagnosis of ER, it has yet to be implemented in clinical practice. Consequently, the potential disease-monitoring benefits of AI in patients with UC remain unclear.

The primary aim of assessing ER is to forecast the risk of future relapse. Although AI-driven relapse prediction using the Ulcerative Colitis Endoscopic Index of Severity [UCEIS] has previously been reported, it was based on only two classifications [presence or absence of ER].^20,21^ Our system is designed to deliver real-time diagnostic results based on video colonoscopy, and is aimed specifically at enhancing endoscopic disease monitoring. This approach contrasts with prior studies that assessed pre-recorded video *ex vivo*.^14,21–24^ Whereas AI offers precise and unbiased diagnostic results, the ultimate responsibility for patient care decisions, including therapeutic interventions, lies with physicians. Therefore, the system allows for saving the target image along with the AI diagnostic output. This enables the endoscopist to document the final diagnosis while reviewing the endoscopic images with attached AI output during report generation.

Our AI system identified ER with a sensitivity of 96.9%, specificity of 78.4%, and accuracy of 93.4%. These results are consistent with those reported in a recent meta-analysis of previously published studies.^25^ Our future efforts will focus on further improving these performance metrics. However, even specialist endoscopic diagnosis, the current gold standard, is subject to variation, and how to determine whether accuracy has improved needs to be established.

A strength of our algorithm is its *in vivo*, real-time output of ‘not a good sample’ to inform endoscopists that the image is ineligible for analysis. In real-world colonoscopy, considerable examination time is spent cleaning residual stool, aspirating intestinal fluid, and adjusting air volume. Hence, we believe that flagging images ineligible for analysis will improve the efficiency of AI-based MES assignment. AI determined that 61.7% [7077/11 472] of the still images taken in this study were ineligible for MES assignment. To ensure accurate AI-based diagnosis, endoscopists must capture high-quality images. Iacucci *et al.* demonstrated that AI provided an accuracy improvement of 2–5% over conventional diagnosis when it used only high-quality videos.^21^ Several previous studies have used similar algorithms to automatically exclude low-quality images from pre-recorded, video-based analysis.^13,22,26^

In this study, among 100 clinically remittent patients, AI predicted that 20% had an MES = 2 or 3. In contrast, clinicians diagnosed Mayo = 2 or 3 in only 11% of cases. This discrepancy can be largely attributed to misinterpretations caused by ineligible images for analysis, such as those containing halos and residual stool, which AI was unable to appropriately exclude despite being unsuitable for analysis. Enhancing the function to identify images ineligible for analysis is anticipated to reduce these discrepancies.

A further strength of our research is the additional diagnostic value of AI-assisted colonoscopy for MES assignment. The results of our study suggest that AI improves the diagnostic accuracy and the intra- and inter-reproducibility of non-specialists. Given considerable variability among even specialists in the assessment of endoscopic severity in UC,^27^ we anticipate that AI can help to reduce their variabilities. However, this sub-analysis was based on pre-recorded video, has not been validated in real time, and did not involve a specialist. Some multi-centre prospective studies using AI to distinguish neoplasia from non-neoplasia have reported that real-time, AI-based validation did not significantly improve diagnostic accuracy.^28,29^ We require further exploration of real-time, AI-based MES assignment to determine whether it can improve diagnostic accuracy of both non-specialists and specialists in UC.

This study had several limitations. First, our system outputs three-class rather than four-class scores. However, in real clinical practice, the management of MES = 2 and MES = 3 remains similar, with treatment intensification being considered in both cases. Second, there was no standard protocol for therapeutic intervention, which was carried out at the discretion of the outpatient physician. Third, the correlation between the diagnostic output of AI and faecal calprotectin levels was not assessed. Fourth, this study was validated in a single centre. To compensate for these limitations, we are currently planning an international, multi-centre trial to assess the potential benefit of AI in relapse prediction by comparing colonoscopies performed with and without AI assistance.

## Conclusion

In conclusion, colonoscopy using AI-based MES assignment can stratify the risk of clinical relapse in patients with UC and may thus aid future disease management.

## Supplementary Data

Supplementary data are available at *ECCO-JCC* online.

jjae080_suppl_Supplementary_Videos_S1-S2_Tables_S1-S2

## Data Availability

Due to the nature of this research, participants of this study did not agree for their data to be shared publicly, so supporting data are not available.
