# Supplementary material for: Artificial Intelligence-assisted Video Colonoscopy for Disease Monitoring of Ulcerative Colitis: A Prospective Study
Source: J Crohns Colitis. 2024 Jun 3;19(1):jjae080. doi: 10.1093/ecco-jcc/jjae080 (PMC11725525; doi:10.1093/ecco-jcc/jjae080)
Supplement: jjae080_suppl_Supplementary_Videos_S1-S2_Tables_S1-S2 [file jjae080_suppl_supplementary_videos_s1-s2_tables_s1-s2.zip › jjae080_suppl_Supplementary Tables_S1-S2 Videos_1-2.docx]

**The detail of selection and creation of videos**

From the videos of the patients in the study, 126 videos ranging in length from 5 to 10 s were generated to present the AI-based diagnostic outcomes (42 videos for each AI diagnosis). To determine the ground truth, three specialists blinded to the AI results provided diagnoses on the basis of the same videos. Diagnoses from all three specialists matched for 82 videos, diagnoses from two specialists matched for 42 videos, and diagnoses from all three specialists differed for two videos. The ground truth for each video was based on the most frequent score assigned by three experienced endoscopists. Hence, the analysis encompassed 124 videos, excluding the two for which the diagnoses from the three endoscopists did not align.

**Supplementary Data 1**

**Video 1**. Artificial intelligence-driven computer-aided diagnosis system for real-time output of three-class scores based on video analysis.

**Supplementary Data 2**

**Video 2.** Artificial intelligence-driven computer-aided diagnosis system for real-time output of “Not a good sample” to inform endoscopists that an image is ineligible for analysis in cases of halation, inadequate air delivery, proximity to mucosa, chromoendoscopy, or virtual chromoendoscopy.

**Supplementary Table**

**Table S1. Mayo endoscopic subscore assigned by non-specialists with and** **without use of artificial intelligence (AI).**

|  | **Without AI** | | |  | **With AI** | | |
| --- | --- | --- | --- | --- | --- | --- | --- |
| **specialists’ label** | **0** | **1** | **2 or 3** |  | **0** | **1** | **2 or 3** |
| **0** | 180 | 63 | 9 |  | 196 | 56 | 0 |
| **1** | 44 | 149 | 29 |  | 46 | 168 | 8 |
| **2 or 3** | 5 | 67 | 198 |  | 1 | 46 | 223 |

**Table S2.** **Comparison of Mayo endoscopic subscore assigned with and without the use** **of AI.**

|  |  | **Without AI** | | |
| --- | --- | --- | --- | --- |
|  |  | **0** | **1** | **2 or 3** |
| **With AI** | **0** | 184 | 53 | 6 |
|  | **1** | 39 | 185 | 46 |
|  | **2 or 3** | 6 | 41 | 184 |
